# Biotransformation of Resveratrol: New Prenylated *trans*-Resveratrol Synthesized by *Aspergillus* sp. SCSIOW2

**DOI:** 10.3390/molecules21070883

**Published:** 2016-07-06

**Authors:** Liyan Wang, Yanhua Wu, Yongtao Chen, Jiaxin Zou, Xiaofan Li

**Affiliations:** 1 Shenzhen Key Laboratory of Marine Bioresource and Eco-environmental Science, College of Life Sciences and Oceanography, Shenzhen University, Shenzhen 518060, China; 13723776301@139.com (Y.W.); cyto2011@126.com (Y.C.); 13691608828@163.com (J.Z.); 2 Shenzhen Key Laboratory of Microbial Genetic Engineering, College of Life Sciences and Oceanography, Shenzhen University, Shenzhen 518060, China

**Keywords:** resveratrol, prenylation, *Aspergillus* sp. SCSIOW2, ECD, erythrocyte protection activity, DPPH scavenging activity

## Abstract

Arahypin-16 (**1**), a new prenylated resveratrol with a unique dihydrobenzofuran ring, has been isolated as a microbial metabolite of resveratrol (**2**) from whole-cell fermentation of *Aspergillus* sp. SCSIOW2. The stereochemistry of **1** was determined by ECD calculations. **1** showed about half of the extracellular radical scavenging effect (IC_50_ = 161.4 μM) compared with resveratrol (IC_50_ = 80.5 μM), while on biomembranes it exhibited the same range of protection effects against free radicals generated from AAPH (IC_50_ = 78.6 μM and 87.9 μM).

## 1. Introduction

Resveratrol is well known for the potential effects on coronary heart disease observed among wine drinkers [1]. Nowadays resveratrol, which possesses a wide range of biological activities, such as antioxidant, anti-inflammatory, antiviral, antimicrobial, anticancer, cardiovascular protection, chemo-protection, neuro-protection and immune-modulation activity is one of the best studied natural products [2]. Previous data have provided interesting insights into the effects of this compound on the lifespan of different organisms and it might become a potential anti-aging agent in degenerative human diseases [3]. However, any further medicinal application of resveratrol is limited due to its low bioavailability and solubility. Researchers have found the addition of isoprenoid substituents on various polyphenol skeletons significantly increased the bioactivity compared to similar compounds that are not prenylated [4]. For example, the introduction of an 8-prenyl group induces estrogenic activity in naringenin. Additionally, 8-prenylnaringenin is rapidly absorbed after oral administration, compared with the generally poor oral bioavailability of flavonoids [5]. Therefore, different strategies have been developed for synthesis of such compounds. The most commonly used organic synthetic strategies are either activated by strong base or by coupling reactions catalyzed by metal salts. Both reactions are usually carried out under extreme conditions and additional steps protection and deprotection are needed. The chemoselectivity, regiochemistry, and number of prenyls are all also difficult to control [6]. Recently, chemo-enzymatic syntheses using enzymes or whole cells were also used for the synthesis of prenylated aromatic components. This type reactions are usually regioselective, stereoselective and occur under mild conditions, thus the introduction of prenyl groups into target compounds by the use of microorganisms represents an attractive alternative to conventional chemical synthesis [7,8]. A series of prenylated resveratrols which have not been previously reported from peanuts has been isolated from peanut seeds challenged by different fungi. These new compounds were considered to play a defensive role against invasive fungi. However, the bioactivities and the real origin of these components were not fully discussed [9,10,11].

**Figure 1:**
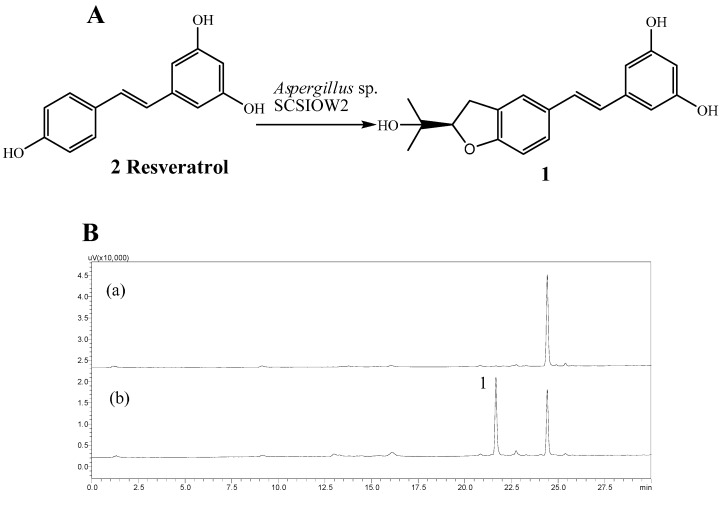
The conversion of *trans*-resveratrol into **1** is catalyzed by *Aspergillus* sp. SCSIOW2. (**A**): Structures of *trans*-resveratrol (**2**) and arahypin-16 (**1**); (**B**): HPLC-UV chromatogram at 309 nm of EtOAc extracts of untreated (**a**) and resveratrol-treated (**b**) fermentation broth.

We are interested in utilizing microbial cultures as biocatalysts to prepare new and potentially active analogues of polyphenolic compounds. In this study, *Aspergillus* sp. SCSIOW2 showed high activity for the regiospecific prenylation of resveratrol. Here we describe the biotranformation, isolation and structure elucidation of arahypin-16 (**1**), a new prenylated resveratrol with an unique dihydrobenzofuran ring, as a microbial metabolite of resveratrol (2) from whole-cell fermentation of *Aspergillus* sp. SCSIOW2 (Figure 1).

## 2. Results and Discussions

A total of 21 fungi strains were screened for their ability to biotransform resveratrol. On the basis of comparative HPLC analyses of test cultures and controls, *Aspergillus* sp. SCSIOW2 was the only organism capable of biotransforming **2** into new metabolites (Figure 1). A preparative scale biotransformation of **2** using whole-cell fermentation afforded metabolite **1**.

**Figure 2:**
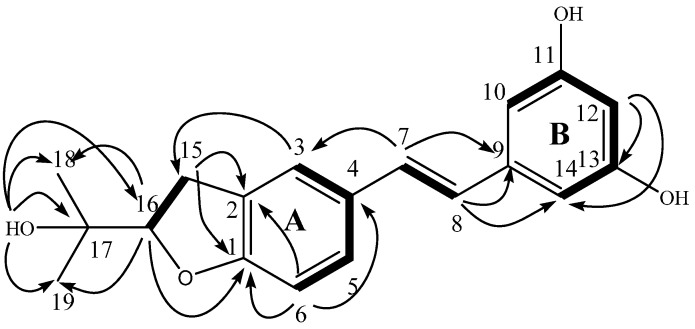
Key ^1^H-^1^H COSY and HMBC correlations of **1** and **2**.

Arahypin-16 (**1**) was isolated as a pale white amorphous powder. The LC-ESI-MS showed a [M + Na]^+^ peak at 335. Using HRESIMS, a molecular ion was measured at 391.0556 (calcd. for C_19_H_20_BrO_4_ [M + Br]^−^, 391.0550), indicating a molecular formula of C_19_H_20_O_4_ with 10 degrees of unsaturation. The ^1^H- and ^13^C-NMR spectra of **1** were similar to those of the previously isolated stilbene arahypin-2, which contained a resveratrol skeleton with a dioxygenated prenyl group at the C-2 position [11]. The ^1^H-NMR spectrum of **1** showed a triplet at δ_H_ 6.11 and a doublet at δ_H_ 6.38 (2H) of an AB2 system for ring B; a singlet at δ_H_ 7.44, a doublet at δ_H_ 7.24, and a doublet at δ_H_ 6.71 for ring A; two coupled doublets at δ_H_ 6.94 and 6.83 for a trans double bond; and a dioxygenated prenyl group (Table 1). However, the mass of **1** was found to be 18 units less than arahypin-2, corresponding to a difference of a H_2_O molecule. Using DMSO-*d_6_* as the solvent allowed also assignments of three hydroxyl protons of **1** (Table 1). Thus, the difference between **1** and arahypin-2 could be the cyclization of the prenyl unit with the oxygen at 16 or 17 position on the 1,2,4-trisubstituted ring. The dihydrobenzofuran moiety was finally established by HMBC correlations from H-16 to C-1 and from 17-OH to Me-18 and Me-19 (Table 1, Figure 2). Finally, careful ^1^H-^1^H COSY, HMQC, and HMBC analyses confirmed the structure as **1** (Table 1, Figure 2).

**Figure 3:**
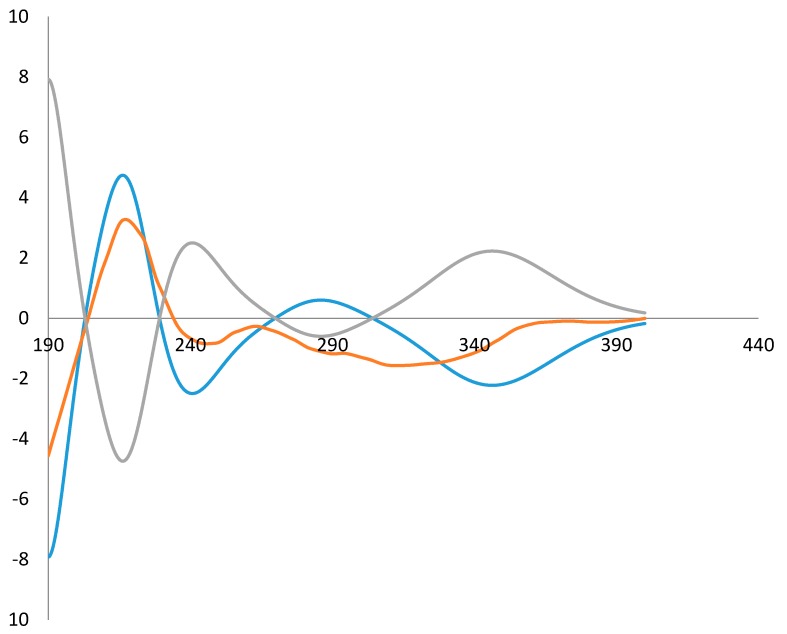
Comparison of the experimental ECD spectrum of **1** (red) with those calculated for the enantiomers 16-(*R*) (blue) and 16-(*S*) (green). (UV correction = 0 nm, bandwidth σ = 0.16 eV).

The ECD spectrum of **1** was then recorded and compared with those calculated for each enantiomer using the time-dependent density functional theory (TDDFT) method [12,13,14,15,16,17]. After conformation space analysis, 22 conformers were found for **1** (Table S1 shows the equilibrium populations of 22 stable conformations in methanol at the B3LYP/aug-cc-pVDZ level). Consequently, the calculated ECD spectrum for the enantiomer 16*R* fit with the experimental plot of **1**, which exhibited two negative and two positive Cotton effects at 315, 245, 263, and 221 nm, respectively. However, the calculated ECD spectrum of the 16-(*S*) enantiomer was opposite to the experimental ECD data (Figure 3). Hence, the stereochemistry of **1** was determined to be 16-(*R*).

**Figure 4:**
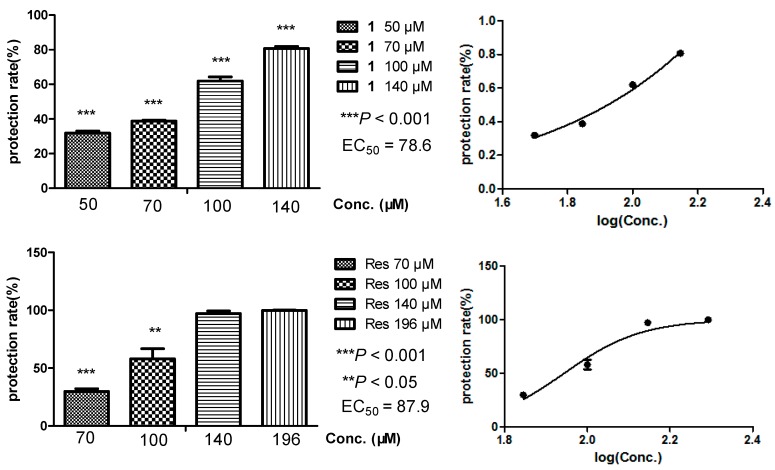
Erythrocyte membrane protection rate (%) of arahypin-16 (**1**) and resveratrol (Res) against hemolysis induced by AAPH.

**Figure 5:**
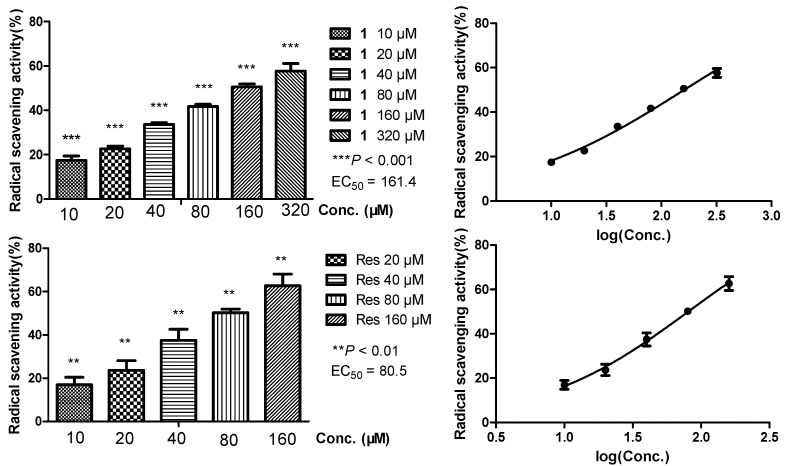
DPPH radical scavenging activity (%) of arahypin-16 (**1**) and resveratrol (Res).

Oxidative damages of cell membranes is closely connected with various free radical-mediated diseases such as rheumatoid arthritis, atherosclerosis, cancer, etc. [18]. Hemolysis of erythrocytes is a relatively simple model for studying oxidative damage to biomembranes due to its enucleated composition [19]. The antioxidant activity of our compounds was evaluated through biomembrane protection against hemolysis induced by 2,2′-azobis(2-methylpropionamidine) dihydrochloride (AAPH), with IC_50_ values of 78.6 μM of **1** and 87.9 μM of **2**, respectively (Figure 4). The DPPH scavenging activity was also assayed to clarify their capacity for scavenging extracellular free radicals, affording IC_50_ values of 161.4 μM of **1** and 80.5 μM of **2**, respectively (Figure 5). Comparing the two experiments, the prenylated compound arahypin-16 (**1**) showed about half the extracellular radical scavenging of resveratrol, while it exhibited the same range of biomembrane protection effect against free radicals produced by AAPH.

Prenylated components are widely distributed in natural products and exhibit a wide range of biological activities. However, in most cases it is not clear whether the prenyl group has a specific role in the bioactivity. The shortage of systematic studies on prenylated components is mainly due to their limited availability. Recently, an impressive number of bioactivities have been demonstrated for prenylated flavonoids, especially for cancer chemotherapy, which has inspired researchers to generate new prenylated components by using different strategies [20]. Chemo-enzymatic syntheses have drawn the attention of scientists, because they are mostly regioselective and stereoselective and occur under mild conditions. Prenyltransferases have been successfully used for the production of more than 250 prenylated derivatives, such as prenylated tyrosine, xanthone, hydroxynaphthalenes, flavonoids, indolocarbazoles, and acylphloroglucinols [6]. In this study, *Aspergillus* sp. SCSIOW2 showed high regiospecific prenylation activity with resveratrol, generating arahypin-16 (**1**), a new component with an unique dihydrobenzofuran ring. This is the first report about the prenylation of resveratrol by fungal biotransformation, which provided us a prenylated resveratrol, arahypin-16 (**1**), for further bioactivity comparison.

## 3. Materials and Methods

### 3.1. General Procedures

Optical rotations were determined on a P-1020 polarimeter (Jasco, Tokyo, Japan). UV data were recorded on a Lambda 25 UV/V is spectrometer (Perkin Elmer, Boston, MA, USA). IR data were recorded using a Nicolet Avatar 330 FT-IR spectrometer (Thermo Scientific, Waltham, MA, USA). NMR spectra were acquired on a Bruker ASCEND 600 MHz NMR magnet system (Bruker, Ettlingen, Germany) using TMS as the internal standard. HPLC-ESIMS was performed using a 6120 Single Quad LC/MS system (Agilent Technologies, Santa Clara, CA, USA). HR-ESIMS was performed using an Bruker maXis). CD spectra were recorded on a Jasco J-815 CD spectrometer. Column chromatography was conducted using silica gel (100–200 mesh, Qingdao Marine Chemical Factory, Qingdao, China) and Sephadex LH-20 (Amersham Pharmacia Biotech, Piscataway, NJ, USA). TLC was performed on Merck TLC plates (silica gel 60 RP-18 F254S and silica gel 60 F254, Merck Millipore Corporation, Darmstadt, Germany), with compounds visualized by spraying with 10% (*v*/*v*) H_2_SO_4_ in EtOH and then heating on a hot plate. HPLC was performed on a Shimadzu LC-20AT pump (Shimadzu Corporation, Tokyo, Japan) equipped with a SPD-20A UV-Vis detector and an Agilent Technologies 1260 Infinity series with a 1260 DAD detector. An Agilent Technologies ZORBAX RX-C18 analytical column (4.6 × 150 mm, 5 μ) was used for analysis and semi-preparative purposes. Resveratrol was purchased from Sinopharm Chemical Reagent Co., Ltd. (Shanghai, China). The experimental New Zealand rabbit was purchased from Guangdong Medical Laboratory Animal Center (animal license: SCXK (Guangdong Province) 2014-0035) and fed at 22 ± 2 °C with relative humidity at 50%. The absorbance measurement in erythrocyte protection assay and DPPH scavenging assay was performed with Varios—Kan Flash (Thermo Fisher Scientific).

### 3.2. Strain

Fungus SCSIOW2 was isolated from a deep marine sediment sample collected in the South China Sea (112°30.203E, 18°1.654N) at a depth of 2439 m. This fungus was characterized as *Aspergillus* sp. Based on the analysis of the ITS region sequence with Genebank S1. This fungus was deposited in the Marine Microbial Lab., College of Life Science, Shenzhen University (Shenzhen, China).

### 3.3. Fermentation, Extraction, and Isolation

Seed medium (10.0% glucose, 2.0% soybean meal, 0.5% peptone, 0.2% NaCl, 0.05% KH_2_PO_4_, 0.024 % MgSO_4_, with the pH adjusted to 7.0). Production medium (7.6% sucrose, 2.6% soybean meal, 1.0% NaOAc, 1.0% citric acid, 0.6% CaCO_3_, 0.5% glycerin, 0.01% MgSO_4_, with the pH adjusted to 7.0). *Aspergillus* sp. SCSIOW2 was cultured in 250-mL Erlenmeyer flasks containing 50 mL of seed medium. After growing at 28 °C, 220 rpm for 3 days, the cellular material was placed in a sterile Falcon tube and mixed by vortexing for several minutes to create a uniform fungal cell/spore suspension. Aliquots (1 mL) of seed cultures were inoculated into 70 mL of production medium in 250-mL Erlenmeyer flasks. At the time of inoculation, 0.5-mL aliquot of EtOH-dissolved resveratrol (16.0 mg) was added, resulting in final concentrations in the liquid media containing 1 mM resveratrol. Control group was added with the same amount of EtOH. The resulting cultures were fermented at 28 °C under static conditions for 7 days. The fermented broth was filtered to obtain supernatant and mycelia. The supernatant broth was extracted three times with 70 mL of EtOAc. The EtOAc extracts of each condition were analyzed by reversed-phase HPLC on a ZORBAX RX-C18 analytical column (4.6 × 150 mm, 5 μ) eluted with MeOH-H_2_O (0:100–100:0 over 30 min, 1.0 mL/min). For preparative scale up, *Aspergillus terreus* was cultivated using 45 250-mL Erlenmeyer flasks containing 70 mL of production medium in the presence of 1 mM resveratrol. The combined EtOAc extract after evaporation (580.0 mg) was applied to a Sephadex LH-20 column chromatograph (CC) with CHCl_3_-MeOH (1:1) to afford seven fractions (Fr.1–Fr.7). Fr.4 (28.6 mg) was further purified by HPLC with a ZORBAX RX-C18 column eluted with MeOH-H_2_O (0:100–100:0 over 30 min, 1.0 mL/min) to yield compound **1** (9.8 mg, t_R_ 19.8 min).

*Arahypin-16* (**1**): white powder; ${\left[\alpha \right]}_{\text{D}}^{27}$ +125° (*c* 1.0, MeOH); UV (MeOH) λmax (log ε) 308 (3.99) nm, 320 (3.98), 218 (3.97), 201 (4.09) nm; IR (film) ν_max_: 3251, 2976, 1594, 1491, 862.7, 833.1 cm^−1^; HRESIMS *m/z* 391.0556 [M + Br]^−^ (Calcd. for C_19_H_20_BrO_4_, 391.0550); ^1^H- and ^13^C-NMR see Table 1.

### 3.4. Quantum Chemical ECD Calculations

In this study, we used the ECD calculation protocol proposed by Nugroho and Morita [13]. The initial 3D structures of the molecules were prepared using Chem3D and minimized with the MMFF94S force field implemented in Chem3D. After the initial structure was further optimized with XedMin in default mode, the conformation space was sampled using XedeX with an energy window of 5 kcal·mol^−1^ above the ground state and RMSD 0.8 to remove duplicated conformers [14]. Then, each conformer was optimized and verified as true minima of the potential energy surface using Gaussian 09 with the DFT method at the B3LYP/aug-cc-pVDZ level [15]. The polarizable continuum model (IEFPCM) was used to take the solvent effects of methanol into account. The optimized conformers were further used to perform a TDDFT calculation at the B3LYP/aug-cc-pVDZ level with the polarizable-conductor calculation model (IEFPCM, methanol as the solvent). In each TDDFT calculation, the 100 lowest electronic transitions were calculated for each conformer. The ECD spectra and overall ECD spectra (weighted by Boltzmann statistics) and comparison of the experimental and calculated spectra were performed using the software SpecDis [16,17].

### 3.5. Preparation of Erythrocytes

Packed erythrocytes were obtained by centrifuging whole rabbit blood with 3.2% citrate at 700 g for 10 min at 4 °C. Cells were washed three times with 0.9% NaCl solution and finally suspended in the buffer solution to obtain a citrated blood, stored at 4 °C and were used within 48 h.

### 3.6. Measurement of Hemolysis and Erythrocytes Membrane Protection

For the hemolysis assay, a method slightly modified from that of Niki, Etsuo, et al. was followed [12,13,21]. Briefly, 6 × 10^8^ mL^−1^ of the washed erythrocytes were pre-warmed at 37 °C and mixed with the assayed compounds dissolved in DMSO and diluted with 0.9% NaCl solution to make final concentrations as described at Figure 4. After incubation at 37 °C for 30 min, 250 µL of AAPH solution at 100 mM was added to induce hemolysis for 60 min at 37 °C. The reaction mixture was centrifuged at 700 g for 2 min and the absorbance of the supernatant was measured at 545 nm. The relative hemolysis was calculated in comparison with the hemolysis in dd water, which was taken as 100%. Each set of experiments was performed in triplicate.

### 3.7. DPPH Radical Scavenging Activity

DPPH scavenging assay was performed as described previously [22]. The assayed compounds were dissolved in DMSO and diluted with mixture of ethanol and 0.4 M HOAc/NaOAc buffer (3:1) to working concentrations, followed with the addition of 20% (*m*/*v*) DPPH ethanol solution, and then incubated in dark for 30 min. The absorbance at 517 nm was measured. DPPH scavenging activity of the samples was calculated in comparison with the absorbance in ethanol and HOAc/NaOAc buffer, which was taken as 100%. All the experiments were carried out in triplicate.

### 3.8. Statistical Analysis

All results were expressed as mean ± SD. Statistical significance (*p* values) of the results was calculated by Student’s *t* test using GraphPad Prism 5 software package (San Diego, CA, USA). The results were considered to be significant when *p* < 0.05.

## 4. Conclusions

In summary, we have reported the conversion of *trans*-resveratrol to arahypin-16 (**1**) catalyzed by *Aspergillus* sp. SCSIOW2. This is the first *trans*-resveratrol derivative containing a dihydrobenzofuran ring and the first report concerning prenylation of resveratrol by direct fungal biotransformation. Arahypin-16 (**1**) showed about half the extracellular radical scavenging of resveratrol, while it exhibited the same range of protective effect on biomembranes against free radicals produced by AAPH. This is also the first report on the biomembrane protection activities of resveratrol and its derivatives.

## Figures and Tables

**Table 1:** NMR spectroscopic data for compound **1** (DMSO-*d*_6_) ^a^.

Position	δ_H_ (Mult, *J* in Hz) ^b^	δ_C_^c^	HMBC
1		160.1	
2		128.7	
3	7.44 s	123.0	C-1, 5, 15
4		129.8	
5	7.24 d (8.4)	127.4	C-1, 3, 7
6	6.71 d (8.4)	109.1	C-1, 2, 4
7	6.94 d (16.2)	128.4	C-3, 5, 9
8	6.83 d (16.2)	126.2	C-4, 7, 9, 10, 14
9		139.7	
10/14	6.38 d (1.8) 2 H	104.7	C-8, 11, 12
11		158.9	
12	6.11 t (1.8)	102.2	C-10, 11, 13, 14
13		158.9	
15	3.13 m	30.2	C-1, 2, 3, 16, 17
16	4.56 t (9.0)	89.7	C-1, 2, 18, 19
17		70.5	
18	1.14 s ^e^	25.2 ^e^	C-16, 17, 19
19	1.12 s ^e^	26.5 ^e^	C-16, 17, 18
11-OH	9.22 s ^d^		
13-OH	9.22 s ^d^		
17-OH	4.61 s		C-17, 18, 19

^a^ Chemical shifts (δ) in ppm; ^b^ 600 MHz; ^c^ 150MHz; ^d^ overlapped signal; ^e^ assignment might be interchangeable.

## Supplementary Materials

The following are available online at http://www.mdpi.com/1420-3049/21/7/883/s1, Table S1: Conformer distribution of **1** in solvated models (methanol) calculation at the B3LYP/aug-cc-PVDZ level, Figures S1–S6: NMR spectra for compound **1**. Figure S1: ^1^H-NMR spectrum of **1** in DMSO-d_6_, Figure S2: ^13^C-NMR spectrum and DEPT of **1** in DMSO-*d*_6_, Figure S3: ^1^H-^1^H COSY spectrum of **1** in DMSO-d_6_, Figure S4: HSQC spectrum of **1** in DMSO-d_6_, Figure S5: HMBC spectrum of **1** in DMSO-*d*_6_, Figure S6: NOESY spectrum of **1** in DMSO-*d*_6_.
