# Supplementary material for: Biotransformation of Resveratrol: New Prenylated trans-Resveratrol Synthesized by Aspergillus sp. SCSIOW2
Source: Molecules. 2016 Jul 6;21(7):883. doi: 10.3390/molecules21070883 (PMC6274042; doi:10.3390/molecules21070883)
Supplement: Supplementary file 1 [file molecules-21-00883-s001.pdf]

Supplementary Materials: Biotransformation of Resveratrol: New Prenylated *trans*-Resveratrol Synthesized by *Aspergillus* sp. SCSIOW2

Liyan Wang, Yanhua Wu, Yongtao Chen, Jiaxin Zou and Xiaofan Li

Table S1. Conformer distribution of **1** in solvated models (methanol) calculation at the B3LYP/aug-cc-PVDZ level.

Conformers	Contribution %
1	5.50
2	4.99
3	4.21
4	4.13
5	3.63
6	3.30
7	2.88
8	2.78
9	2.78
10	2.43
11	2.35
12	2.33
13	2.29
14	2.29
15	2.27
16	2.23
17	2.00
18	1.97
19	1.90
20	1.85
21	1.84
22	1.82

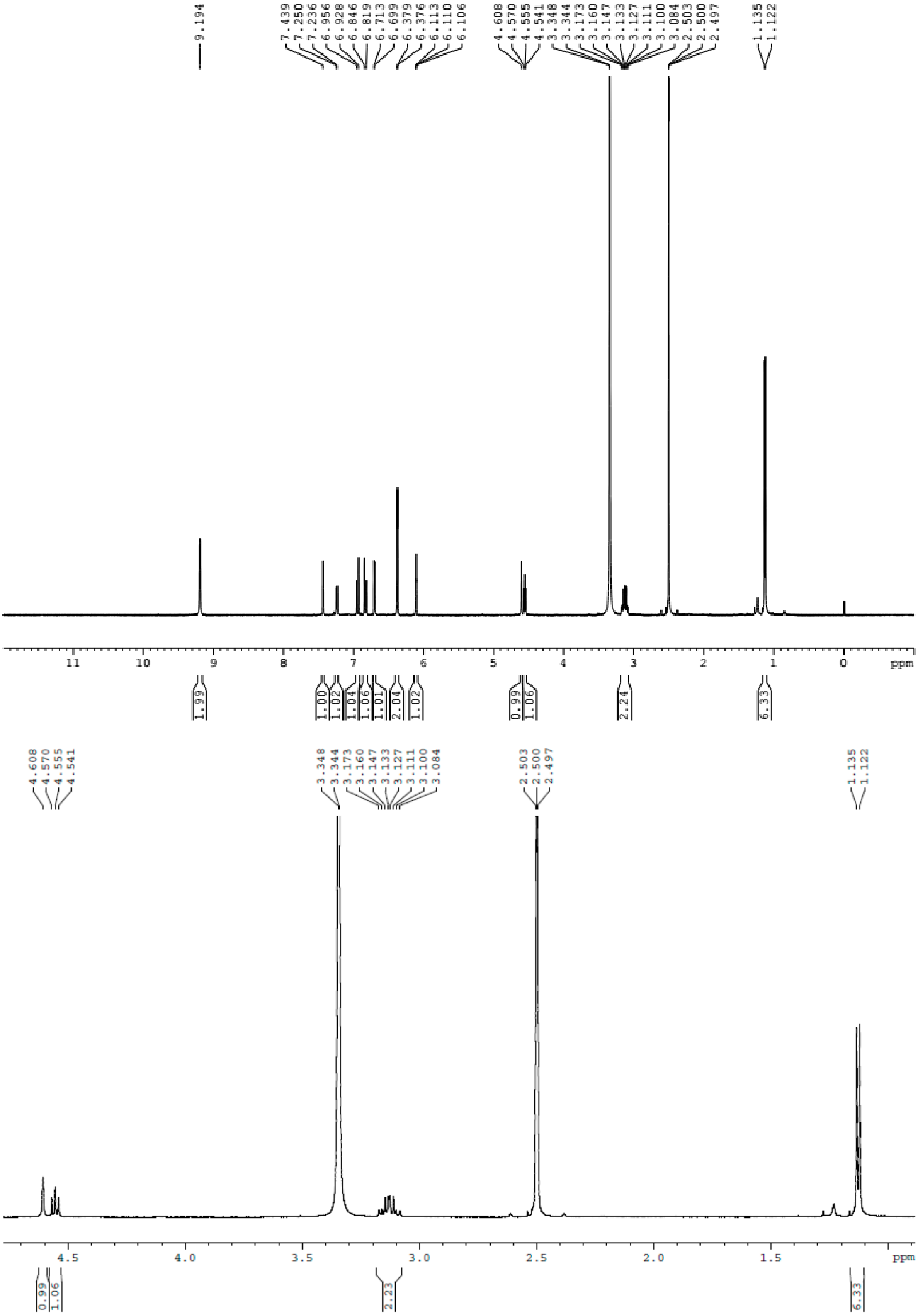

Figure S1. *Cont.*

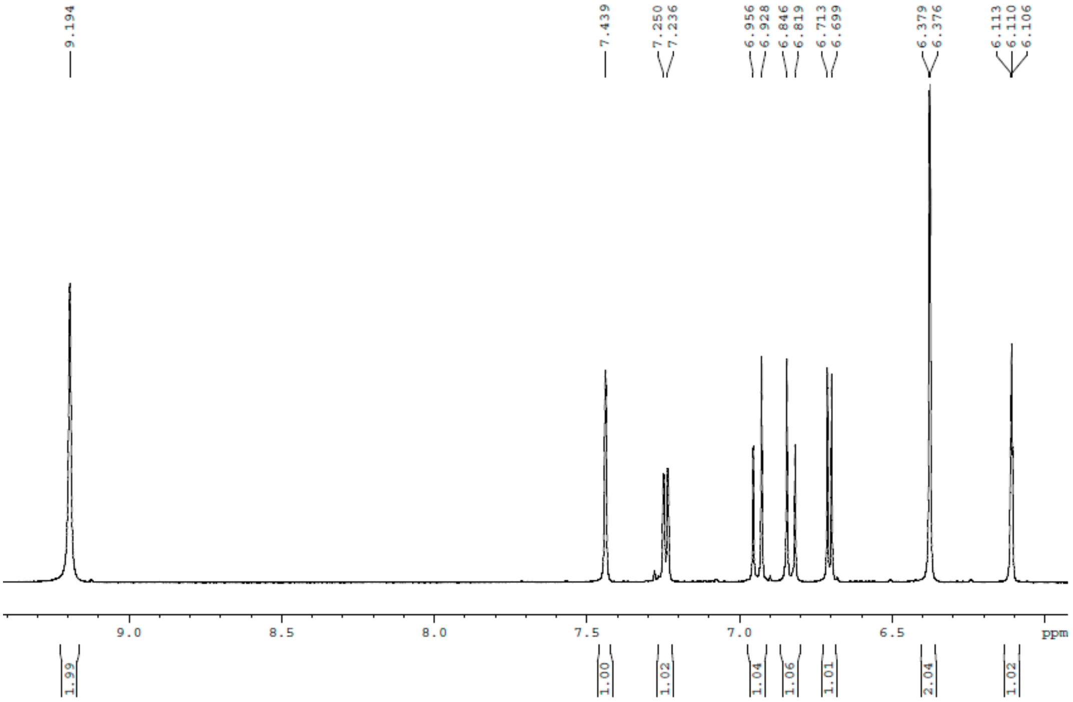

Figure S1. ^{1}H-NMR spectrum of **1** in DMSO-d_6.

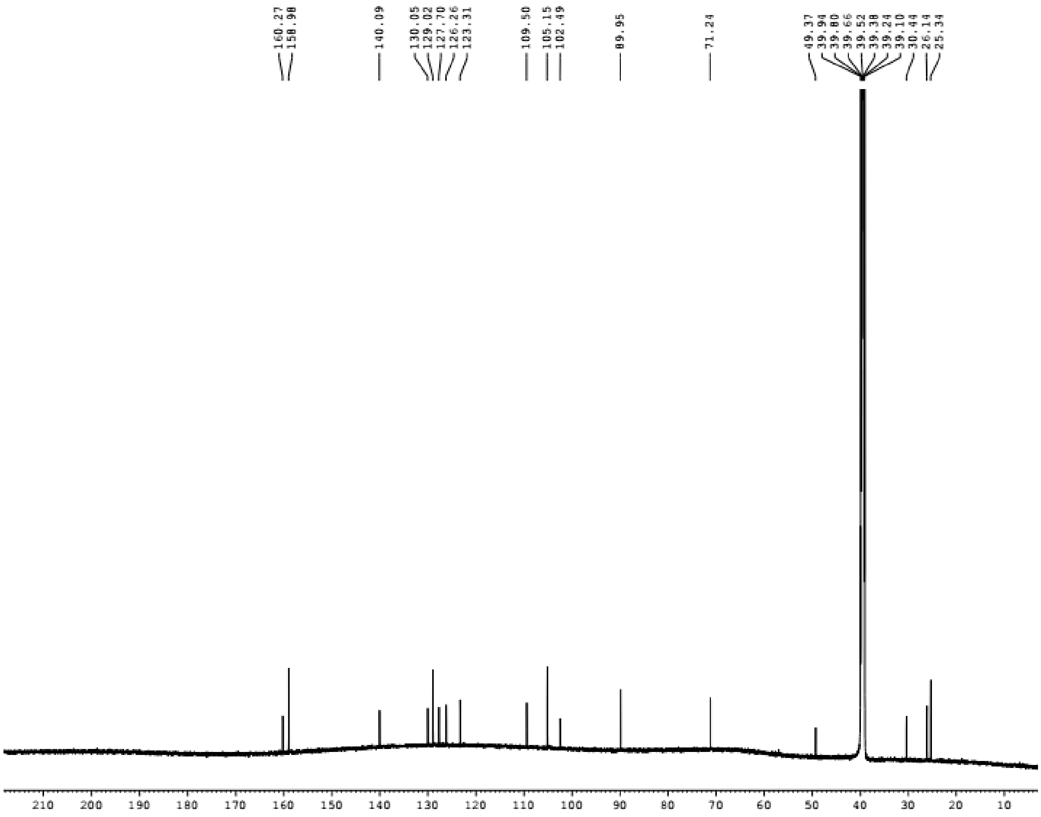

Figure S2. *Cont.*

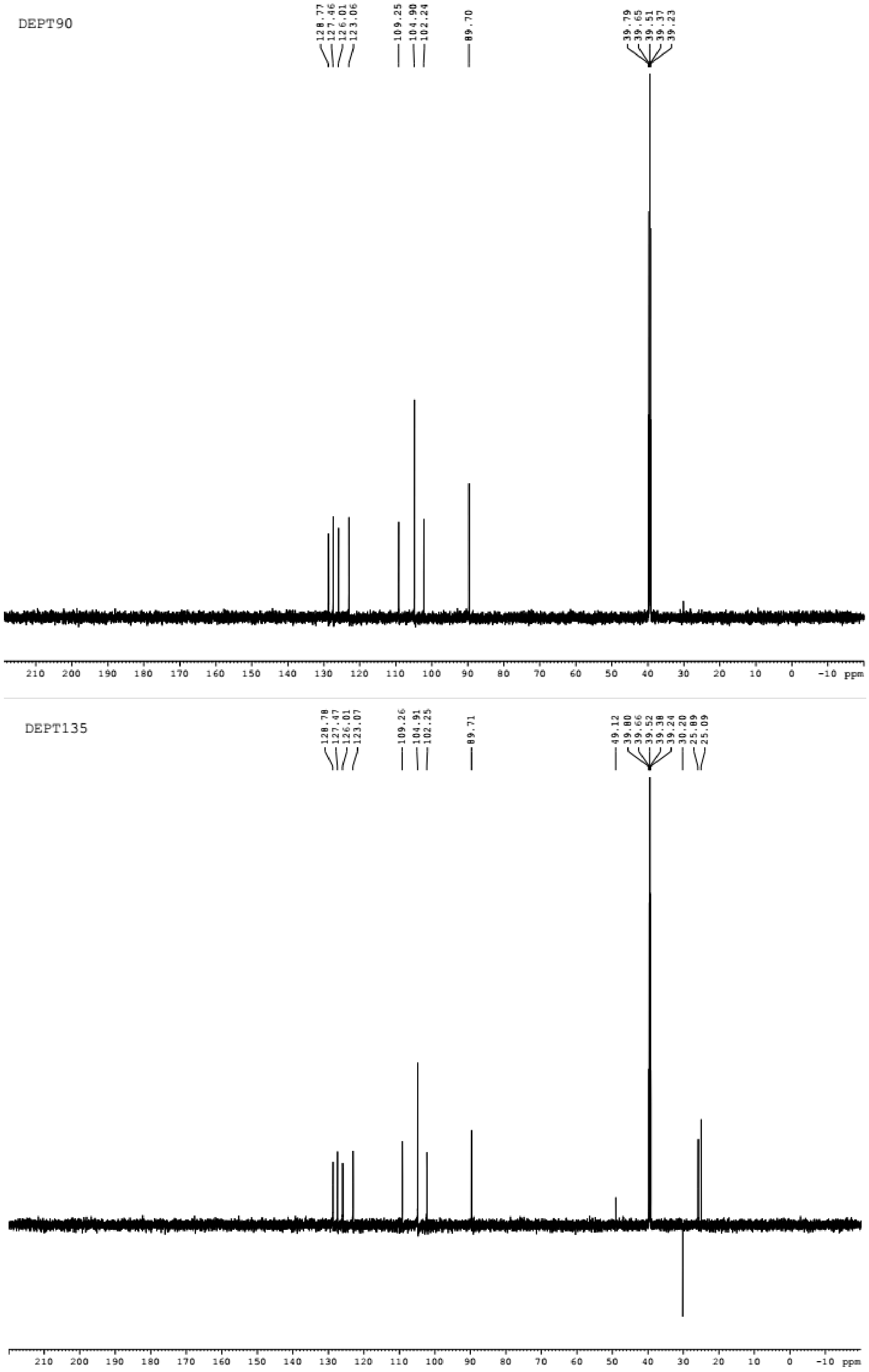

Figure S2. ^{13}C-NMR spectrum and DEPT of **1** in DMSO-d_6.

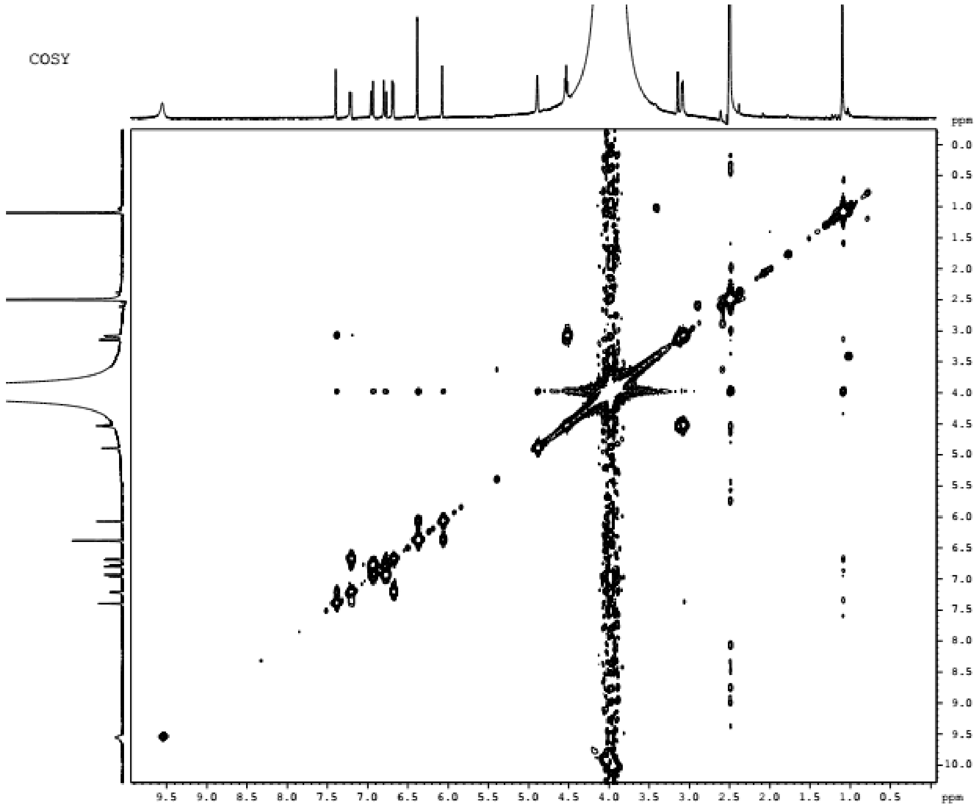

Figure S3. ^{1}H-^{1}H COSY spectrum of **1** in DMSO-*d*6.

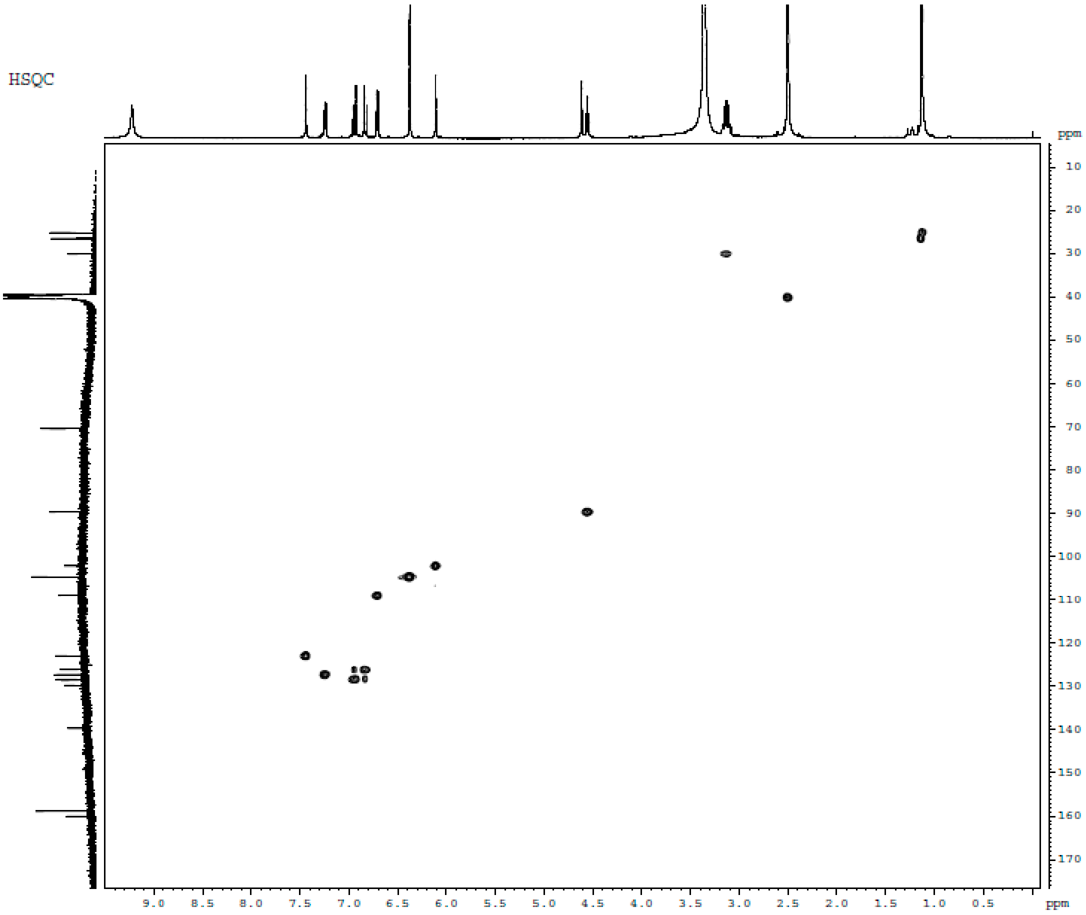

Figure S4. HSQC spectrum of **1** in DMSO-*d*6.

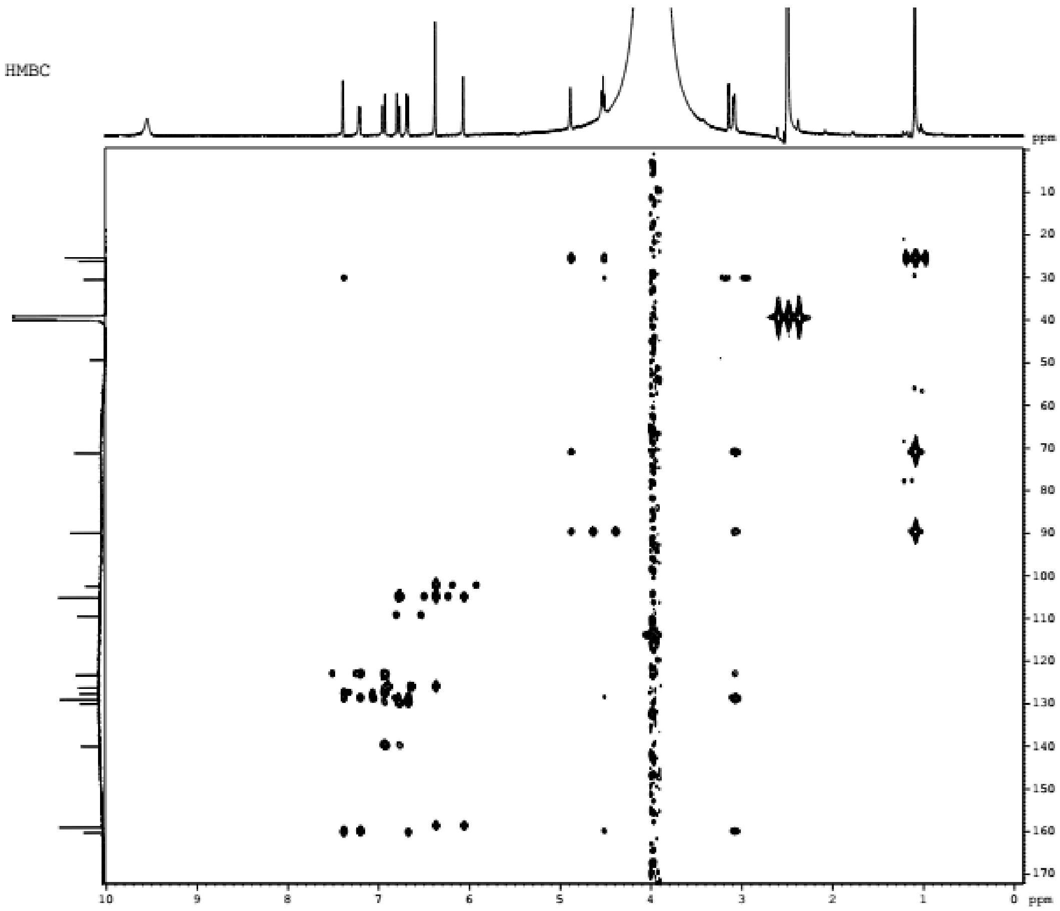

Figure S5. HMBC spectrum of **1** in DMSO-d_6.

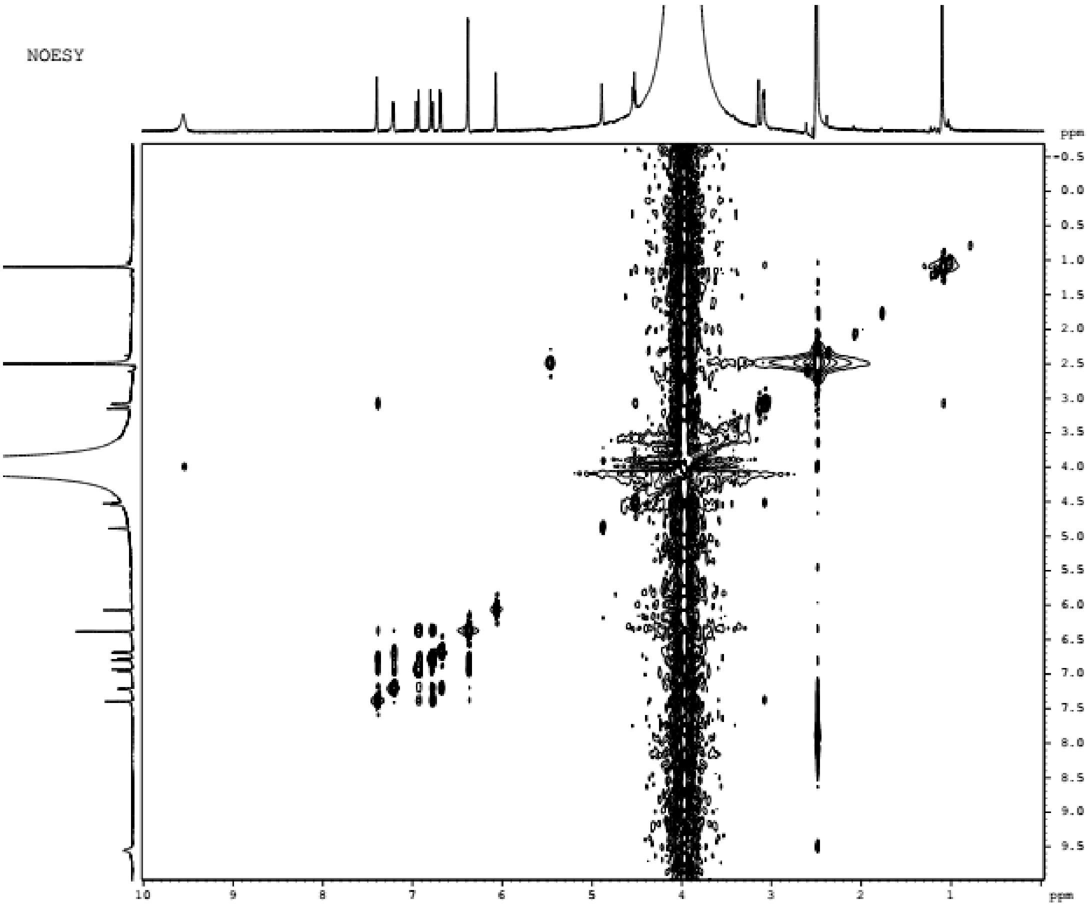

Figure S6. NOESY spectrum of **1** in DMSO-d_6.
